# Discriminating the Difference between Remote and Close Association with Relation to White-Matter Structural Connectivity

**DOI:** 10.1371/journal.pone.0165053

**Published:** 2016-10-19

**Authors:** Chinglin Wu, Suyu Zhong, Hsuehchih Chen

**Affiliations:** 1 Department of Educational Psychology and Counseling, Taiwan Normal University, Taipei, 10610, Taiwan; 2 State Key Laboratory of Cognitive Neuroscience and Learning & IDG/McGovern Institute for Brain Research, Beijing Normal University, Beijing, 100875, China; Plymouth University, UNITED KINGDOM

## Abstract

Remote association is a core ability that influences creative output. In contrast to close association, remote association is commonly agreed to be connected with more original and unique concepts. However, although existing studies have discovered that creativity is closely related to the white-matter structure of the brain, there are no studies that examine the relevance between the connectivity efficiencies and creativity of the brain regions from the perspective of networks. Consequently, this study constructed a brain white matter network structure that consisted of cerebral tissues and nerve fibers and used graph theory to analyze the connection efficiencies among the network nodes, further illuminating the differences between remote and close association in relation to the connectivity of the brain network. Researchers analyzed correlations between the scores of 35 healthy adults with regard to remote and close associations and the connectivity efficiencies of the white-matter network of the brain. Controlling for gender, age, and verbal intelligence, the remote association positively correlated with the global efficiency and negatively correlated with the levels of small-world. A close association negatively correlated with the global efficiency. Notably, the node efficiency in the middle temporal gyrus (MTG) positively correlated with remote association and negatively correlated with close association. To summarize, remote and close associations work differently as patterns in the brain network. Remote association requires efficient and convenient mutual connections between different brain regions, while close association emphasizes the limited connections that exist in a local region. These results are consistent with previous results, which indicate that creativity is based on the efficient integration and connection between different regions of the brain and that temporal lobes are the key regions for discriminating remote and close associations.

## Introduction

Remote association is the ability to link concepts that were relatively remote into new relationships and to fulfill special needs or goals, i.e. creativity [1]. Creativity can improve the value of existing things, and thus promote the development of human civilization and science [2, 3]. Mednick described the difference between people with high remote association abilities and others on an association hierarchy [1]. High remote association ability people whose flat organization of semantic memory structure could link a greater number of concepts and gradually links them to remote concepts; less remote association ability people with steep association, connect more close concepts instead of remote concepts. In particular, remote concepts are the knowledge nodes that are distant and less relevant to the topics in a semantic network; close concepts are close and highly relevant nodes [4].

To evaluate an individual’s remote association ability, Mednick [5] developed the Remote Associates Test (RAT), which was based on his association hierarchy. The RAT was composed of common and usual verbal material; the researchers selected three words as stimuli from the verbal norm that were mutually remote to one another, and the participants needed to find an intervening word. The RAT is economic to use and easy to score, and it has been generally used in creativity research [6, 7, 8, 9]. It was first adapted for Mandarin speakers by [10]. They used the method of semantic compounding that Mednick [5] used, and they developed the items of RAT by word-pairings and built the Chinese Remote Associates Test (CRAT). Every item had three stimulus words, for example, “生” (to generate), “天” (sky), and “溫” (warm). The participants were asked to find an intervening word for each of the three stimuli words and to compose an actual two-word “target word”, in this case, “氣” (air), to “生氣” (anger), “天氣” (weather), and “氣溫” (temperature). The CRAT had 30 items and had good criterion-related validity to the Creativity Assessment Packet.

Remote association has precise definition and method of measure [1, 5], however, close association has fewer research. By the perspective of problem solving process, when remote associates task, individual would initiated by one of the stimuli to link to every possible words, and cross-checked result of association with other two stimuli to confirm the potential answer [11]. If target words were remote concept, it’s harder to link; in contrary, if target words were close concept, it’s easier to connect. Therefore, if all three stimuli were remote concept, individuals still could link to target word which was relative remote to the stimulus word but linkable to other two stimuli words, it is the remote association performance. It is similar to the concept of creative synthesis [12] which emphasized the integration of divergent thinking and convergent thinking to develop creativity. People developed all potential answers via divergent thinking and pointed to one best answer by convergent thinking [13]. Besides, if all items are close concept, the performance during the task is only about close association. It also requires convergent thinking to find best answer but does not need divergent thinking to connect the close concept. Therefore, we know that remote association and close association have different mechanism. Empirical study indicated remote association took more response time to initiate [14], it supported the concept that remote concept located at relatively distant place than close concept in semantic network. Moreover, when remote associates task, it would be harder to link correct answers if individuals linked toward high frequency words [15], it refers that remote association needed to connect to distant concepts. Meanwhile, high frequency words and close concepts are somewhat similar, both refer to the content that is easier to be thought of, generating more close concepts does not benefit the development of creativity. In sum, the process of remote associates test might differ due to component of items (e.g. item difficulty, linkage strength of stimuli and target), thus there are remote association and close association. Behavioral study indicated remote association test performances composed of high and low frequency words were different [16], but we still don’t know yet the connection between close/remote association and brain structure.

Neuroimaging research has indicated that the process of creativity can be observed by many brain activities [17]. Currently, no brain study focused on remote association, therefore, we refer to the brain image research for creativity which is strong related to remote association, so that we could presume the potential brain operation during remote association. The fMRI results showed that when people were working on novel and creative ideas, brain activations were found in the middle temporal gyrus, anterior cingulate, and the superior and inferior parietal lobules [18, 19]. In addition to fMRI research, other studies have also investigated the correlation between personal behaviors and sMRI, and they attempted to find the connection between the brain and behavior. The resting state brain images showed verbal creativity positively correlated with the thickness and volume of the right precuneus but negatively correlated with regional homogeneity (ReHo) of the right precuneus. Additionally, originality had a negative correlation with the ReHo of the left superior frontal gyrus, but a positive correlation with the right occipito-temporal gyrus [20]. Furthermore, the composite creativity index (CCI) which reflects the divergent thinking performance had a negative correlation with the cortex thickness of the lingual gyrus but a positive correlation with the right posterior cingulate; creative achievement had a positive correlation with the thickness of right angular gyrus but a negative correlation with the volume of the left lateral orbitofrontal [21]; and divergent thinking had a medium and positive correlation to the functional connectivity of the medial prefrontal cortex and posterior cingulate [22]. A diffusion tensor imaging (DTI) study showed that divergent thinking and the fractional anisotropy (FA) of white matter had a positive correlation and that people who had better divergent thinking had stronger connections among the brain regions [23]; CCI had a negative correlation with FA of the white matter of the left inferior frontal lobe [21].

In recent years, researchers have come to view the brain region connections as a complicated network [24, 25] and indicated that creativity was a result of the interaction between the default network and the control network of the human brain [26, 27, 28]. The development of creative ideas had a positive correlation to the functional connection between the inferior prefrontal cortex and the default network [26]. The global efficiency of the divergent thinking network and CCI had a positive correlation [27]. In addition, some researchers used DTI (diffusion tensor imaging) tracking of the water diffusion of white matter and described the brain network structure of brain tissues and neural fibers [29], indicating the clustering level and connection efficiency of the brain regions using some indices of graph theory, such as the clustering coefficient and the characteristic path length [30, 31]. The graph theory approach treated the connections among the brain regions as complicated networks, and it defined “brain region” as a node and “functional connection among brain regions” as the side length; mathematical formulas were used to build a network, and the researchers calculated the attributes of the network and nodes, such as the clustering coefficient, characteristic path length, global efficiency, local efficiency, regional efficiency, and small-worldness [30, 32]. There are already some studies that have investigated the correlation among creativity, intelligence, gender, and the structure of white matter using graph theory [33, 34, 35], which found that divergent thinking ability and attributes had a negative correlation in the female group but no significant finding in the male group. This finding indicates that information delivery among the brain regions costed more when females had novel and unique thoughts.

Reviewing past neuro-cognition research about creativity, there was still no study that investigated the difference between remote association and close association from the perspective of brain operation. Understanding the similarity and difference between remote and close associations will help us to find why some people have creative outlets with remote association. To address this issue, the present study aims to analyze the brain network structure and the connection efficiency of the nodes between different distance associations. Based on the previous results using DTI [23, 33] and neural brain imaging of creativity [18, 19, 26, 27, 28], the present study hypothesizes that remote association positively correlates with the overall connection efficiency of the brain network and has a positive correlation with the connection efficiency in the temporal lobe; however, it is negatively correlated and has a negative correlation with respect to close remote association.

## Materials and Methods

### Participants

35 adults (18 males and 17 females) with normally functioning nerves volunteered to participate in this study. The participants were 20 to 30 years of age (*Mean* = 23.63, *SD* = 2.62) and native Chinese speakers. They had no obvious reading ability disorders in Chinese. In addition, they did not consume any alcohol for 24 hours before the experiment. Approval for the research was obtained from the Behavioral and Social Science Research Ethics Committee of National Taiwan University. All of the participants understood the research content and signed the informed consent declaration before the study began.

### Measures

This study used the remote and close associates test and the Wechsler Adult Intelligence Scale (WAIS) as tools. The descriptions are as follows:

#### Remote and close associates test

The remote associates test [5] is composed of common languages materials. Each item contains three mutual remote stimulus words, and requires the participants to find an intervening word that mutually connects the three stimulus words. For example, for the stimulus words of “blood”, “music” and “cheese”, the answer is blue, which forms the phrases of blue blood, blue music, and blue cheese.

The present study borrows the design of RAT by Mednick [5] and extends the development of the present CRAT [10] to edit CRAT word-pair items. Each item contains three stimuli, for example, “今” (now), “輕” (light), and “去” (last), and the participants attempt to find an intervening word as a target that could be paired with the three stimuli to create three actual words; the answer is “年” (year) (“今年” (this year), “年輕” (young), and “去年” (last year). With regard to the selection of material, this study used the Statistical Report on Word Frequency compiled for the Material Edition of the Concise Mandarin Chinese Dictionary from the Ministry of Education as the source for compiling the test items and referred to the development of the CRAT [10, 36]; the present study used extreme groups and defined close concepts as phrases whose occurrence frequencies ranked in the top 1/3 of all phrases, and those that ranked in the bottom 1/3 of all phrases were defined as remote concepts. More specifically, high-frequency word means people have more opportunity to link, and vice versa. At the same time, the onomastic on in different domains is driven out in order to prevent the background knowledge which belongs to specific domain will influence the respondents' answering questions. According to this criterion, this study select remote associative vocabularies (the mean frequency is 2.38, *SD* = 0.54) and close associates vocabularies (the mean frequency is 32.58, *SD* = 20.57). This study designed 40 items each for both remote and close association tests, a total of 80 items. Statistical test confirmed that the number of occurrences for these two groups of items reached a significant difference (*t* (78) = 16.07, *p* < .001). In addition, to ensure that the participants have an association space to link the remote concepts and close concepts while problem solving, the present study selected target words that had at least 20 actual associations with stimulus words (i.e., the first to third ranking of association words among all the words) and controlled the frequency of the linking words with the average of each stimulus (Mean = 31.65). Last, according to previous study, forward association facilitated correct rate [37] and avoid the response set by the association direction, the present study controlled the directions of association to be 3:5 (45 forward association, 75 backward association) to increase the difficulty. It also randomly allocated the stimulus words to actual words, to make it possible to have a response set.

#### Wechsler Adult Intelligence Scale-III (WAIS-III)

This study used the Chinese version of the Wechsler Adult Intelligence Scale translated by Chen and Chen [38], including the verbal scale and the performance scale. There was a total of 14 test subjects: vocabulary, picture completion, similarities, digital symbol coding, arithmetic, graph designs, memory span, matrix reasoning, common information, serial graphics, comprehension, symbol search, character-number sequencing, and object and model matching, with the last three subjects being substitution tests. The split-half reliability varied from .89 to .98, and the test-retest reliability for time intervals from two weeks to eleven weeks varied from .86 to .97. Using the Wechsler Adult Intelligence Scale (Revision) (WAIS-R) as the criterion test, the concurrent validity varied from .86 to .94. This study was norm-referenced in Taiwan.

Because it was commonly agreed that the verbal abilities of the participants have a significant influence on remote associative tests, this study used only the verbal scale of the Wechsler Adult Intelligence Scale as the control variable when assessing the structural properties of the brain network and the ability to perform remote associations.

### Procedure

The participants answered with computers and pen-and-pencil. Before the beginning of the experiment, the experimenter fully explained the goal and task content, and the participants signed the informed consent and then completed “Remote and Close Associative items”, “Wechsler Adult Intelligence Scale-III”, and brain imaging scanning.

First, we used the computer screen to display the “Remote and Close Associates Items”, and the computer screen displayed the items one by one; each interval contained 16 items, there was a total of 5 intervals, and the length of the test was 80 items. There was a break for 3 minutes after each interval. An item was on screen for 20 seconds. When the participants thought of the answer within the time-limit, they could press the button to enter the answer page and wrote down the answers in the answer sheet. The participants moved to the next item when they finished the present item. If they failed before the time limit, they had to pass the present item and moved to the next item.

Next, the participants answered the subscales of vocabulary, similarities, arithmetic, memory span, common information, comprehension, and character-number sequencing for the Wechsler Adult Intelligence Scale-III. It took approximately 40 to 50 minutes. After finishing the behavioral tasks, the participants took a 5 minute break. Then, they moved to brain imaging scanning, including the sMRI and DTI; the scanning time was approximately 20 minutes.

### MRI acquisition

This study conducted MRI scanning at the Taiwan Mind and Brain Imaging Center, National Chengchi University. The device used was a Siemens high-field MRI, with a head coil that has 32-channel 90-degree phase differences and input/output functions.

First, a T1 anatomical image scan was conducted, which was a T1-weighted structural scan. It was targeted on the activated and connected brain regions. The pulse sequence of the structural image was 3D-MPRAGE. The related scanning parameters were set as follows: TR = 2530 ms, TE = 3.30 ms, FA = 7°, frame resolution (matrix size) = 256 × 256 mm^2^, FOV = 256 × 256 mm^2^, slice number = 192, thickness = 1 mm and spatial resolution = 1 × 1 × 1 mm^3^.

Next, a diffusion tensor imaging scan was conducted to trace the activities of the water molecules in the brain fiber tracts. The scanning parameters were set as follows: TR = 11000 ms, TE = 98 ms, 30 optimal nonlinear diffusion weighting directions with b = 1000 s/mm2 and five additional images without diffusion weighting (i.e., b = 0 s/mm2); b-value = 1000 s/mm^2^, matrix size = 128 × 128 mm^2^, FOV = 224 × 224 mm^2^, slice number = 70, thickness = 1.8 mm, and spatial resolution = 1.8 × 1.8 × 1.8 mm^3^.

### Data preprocessing

The preprocessing of the image information includes removing the images of the skull and other non-brain materials [39]. This study corrected and readjusted the deviations caused by the head movements of the participants and the scanning distortion; fitted and decomposed the local diffusion tensor; and calculated the fractional anisotropy index. The researchers used PANDA (pipeline tool for diffusion MRI) [40] to conduct preprocessing of all of the image information. This software can utilize related tools, such as FMRIB Software Library (FSL) [41], Pipeline System for Octave and Matlab (PSOM) [42], Diffusion Toolkit [43], and MRIcron (http://www.mccauslandcenter.sc.edu/mricro/mricron/).

### Construction of binary white-matter connectivity networks

In this study, the automated anatomical labeling (AAL, [44]) atlas was used to segment the cerebral cortex of each subject into 90 regions (45 for each hemisphere) without the cerebellum. Each region represents a node of the DTI-based WM network. The detailed parceling processes were implemented according to the procedure proposed by Gong and colleagues [25]. Briefly, the T1-weighted image was first non-linearly normalized to the MNI space. Next, the fractional anisotropy image of each subject was co-registered to the individual T1-weighted image. Finally, the inverse transformations from the previous two steps were applied to the atlas, which resulted in native-space GM parcellations for each subject. The deterministic fiber assignment continuous tracking (FACT) algorithm was applied to reconstruct whole-brain WM tracts [45] using the Diffusion toolkit (http://trackvis.org), which is embedded in PANDA [40]. Specifically, the tracking procedure terminated if the turn angle of the fiber was greater than 45° or the fiber entered a voxel with a fractional anisotropy of less than 0.2. Two region pairs, A and B, were considered to be structurally connected (i.e., having an edge) if there existed at least three tracts with terminal points in both regions A and B [46]. Combining the above definitions of the nodes and edges, we attained for each subject a 90⊆90 binary network whose elements indicated the existence/absence of an edge between any pair-wise regions.

### Network analysis

This study adopted graph theory measures to calculate the global and local connectivity efficiencies of the brain network and the small-world attribute of the integrated network. Indices for evaluating the network efficiencies included the clustering coefficient (*C*_*p*_), characteristic path length (*L*_*p*_), normalized Cp (λ), normalized Lp (γ), small-worldness (ζ), global efficiency (*E*_*glob*_), local efficiency (*E*_*loc*_), and nodal efficiency (*E*_*nodal*_). These graph-theoretical network metrics were calculated by using the in-house GRETNA package [47].

#### Clustering coefficient (*C*_*p*_)

The clustering coefficient is used to evaluate the clustering levels of each of the network nodes. It represents the possibility of mutual neighboring states between the neighboring nodes of a node. The clustering coefficient equals the ratio of the number of actual connections to that of the possible maximal connection sides among the neighboring nodes of a node.

#### Characteristic path length (*L*_*p*_)

The characteristic path length refers to the optimal route for signals to transmit from one node to another in the network. Through the shortest path, the signals can be delivered faster, which saves the resources of the system. The shortest path between two nodes is the path that travels through the least number of nodes. The length of the shortest path of the network is the mean value of the shortest path length between any two nodes in the network. It is calculated by the following equation:$L_{p}=\frac{1}{N\left(N-1\right)}\Sigma_{i\;\in G}\Sigma_{j\;\in G}\frac{1}{L_{ij}}$.

#### Small-worldness (ζ)

The concept of small-worldness was proposed by Watts and Strogatz [32]. When the transmission between the nodes of a network has the shortest path length and the optimal clustering coefficient, this network is considered to possess the property of small-worldness [32]. The judging standard is that $\zeta =\frac{\gamma }{\lambda }\;>\;1$, where γ is the normalized clustering coefficient, which is the ratio between the clustering coefficients of the actual network and the random network (Cpreal / Cprandom). Here, λ is the normalized path length, which is the ratio between the path length of the actual network and the random network (Lpreal / Lprandom). When λ is larger than one and γ is close to 1, ζ will be larger than 1, which means that the network has the small-world attribute. The larger the value of ζ is, the stronger the small-world attribute of the network.

#### Global efficiency (E_glob_)

The global efficiency examines the transmission efficacy among the nodes of the entire network. It is obtained by calculating the harmonic mean of the path lengths among all of the nodes. The calculation equation is $E_{glob}=\frac{1}{N\;\left(N-1\right)}\Sigma_{i\neq j\in G}\frac{1}{L_{ij}}$. The larger the figure is, the higher the global transmission efficiency of the entire network.

#### Local efficiency (E_loc_)

The local efficiency is the extension of the concept of the clustering coefficient. Scholars improved the problem whereby the clustering coefficient considered only the nodes that were directly connected to a node, extending the idea to calculate the connectivity conditions near this node. The local efficiency of the network is the mean of the efficiencies of all of the nodes. It is calculated by $E_{loc}=\frac{1}{N}\Sigma_{i\in G}E_{glob}\left(G_{i}\right)$, where Gi is the secondary network that is constructed by the neighboring nodes of node i.

#### Nodal Efficiency (E_nodal_)

The nodal efficiency is a measure of the nodal capacity to communicate with other nodes in the network. The nodal efficiency for a given node (*E*_*nodal*_) was defined as the inverse of the harmonic mean of the shortest path length between the node and all of the other nodes in the network: $E_{nodal}\left(i\right)=\frac{1}{N-1}\Sigma_{i\neq j\in G}\frac{1}{L_{ij}}$, where *L*_*ij*_ is the characteristic path length between node *i* and node *j*.

### Statistical analysis

To discuss the correlation situations between an individual’s ability for remote and close association and the organization connectivity of the brain network, this study calculated the related indices of the connectivity efficiencies of the brain network and took the participant’s age, gender, and verbal intelligence as the control variables. This study calculated the correlation between the efficiencies of the network node and the scoring for remote and close association to further examine their correlation for the 90 brain regions.

## Results

### Behavioral data

Table 1 shows the mean scores and SD of the participants of different genders in the test of verbal intelligence, remote and close association. According to the results, the mean correct rates for the remote and close association were 0.18 (SD = 0.08) and 0.28 (SD = 0.11), respectively. The mean score for the verbal intelligence test was 116.77 (SD = 7.44). In particular, the correct rate of close association was significantly higher than that of the remote association (*t* (34) = 3.59, *p* = .001, *d* = 1.23). None of the background variables (gender, age, and verbal intelligence) showed significant correlations to the remote and close association (*rs* < -.16, *ps* > .34).

**Table 1:** Demographic Statistics for the Sample of Males and Females.

Measures	Male (*N* = 18)		Female (*N* = 17)	
	*Mean*	*SD*	*Mean*	*SD*
Age	23.29	2.56	23.44	2.76
Verbal Intelligence	116.56	6.78	117.00	8.29
Remote Association Test	0.18	0.09	0.18	0.08
Close Association Test	0.27	0.11	0.29	0.11

### Small-world properties of brain networks

The structures of the brain networks of all of the participants showed small-worldness, which means that compared to a random network, the brain network has a shorter path length and a higher clustering coefficient (ζ = 5.79 ± 0.75), which indicates that regardless of whether there are high or low individual abilities for remote association, the brain network has a good topological properties.

### Correlations between remote and close association and brain network properties

The analysis results are shown in Table 2. After taking the gender, age, and verbal intelligence of the participants as controls, remote association showed significantly positive correlations to the global efficiency (*r* = .43, *p* = .015) and normalized path length (*r* = .40, *p* = .022) and showed significantly negative correlations to small-worldness (*r* = -.40, *p* = .024) and the normalized clustering coefficient (*r* = -.36, *p* = .046). On the other hand, close association showed a significantly positive correlation to the shortest path length (*r* = -.44, *p* = .012) and significantly negative correlations to the global efficiency (*r* = -.44, *p* = .012) and the normalized clustering coefficient (*r* = -.51, *p* = .003).

**Table 2:** Correlation Coefficients of Remote, Close Association and Brain Network Properties (*N* = 35).

	*C*_*p*_	*L*_*p*_	*E*_*loc*_	*E*_*glob*_	ζ	γ	λ
Remote Association	0.07	-0.34	-0.03	0.43*	-0.40*	-0.36*	0.40*
Close Association	-0.15	0.44*	0.08	-0.44*	0.25	0.15	-0.51**

*p < .05,

**p < .01

Note: C_p_: clustering coefficienct, L_p_: characteristic path length, E_loc_: local efficiency, E_glob_: global efficiency, ζ: small-worldness, γ: normalized characteristic path length, λ: normalized clustering coefficienct.

**Fig 1:**
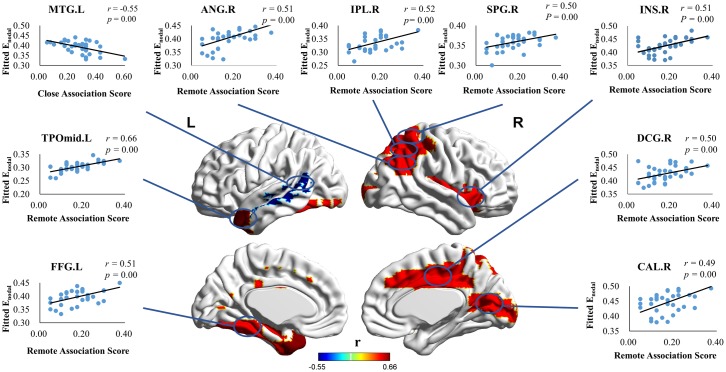
The Spatial Distribution of Cortical Regions Showing Significant Associations between the Nodal Efficiency, Remote and Close Association Scores. R: Right hemisphere; L: Left hemisphere; TPOmid: Temporal pole: middle temporal gyrus; IPL: Inferior parietal, but supramarginal and angular gyri; INS: Insula; DCG: Median cingulate and paracingulate gyri; FFG: Fusiform gyrus; ANG: Angular gyrus; CAL: Calcarine fissure and surrounding cortex; SPG: Superior parietal gyrus; MTG: Middle temporal gyrus.

This study further analyzed the correlations between remote and close association and the global efficiencies of all of the brain nodes after excluding the influences of gender, age, and verbal intelligence. The study adopted the corrected α value after multiple comparisons (1/90) as the standard for significance. Table 3 lists the results, which show that remote association has significantly positive correlations to the connectivity efficiencies of certain brain regions, including the left middle temporal gyrus (*r* = .66, *p* = .001), right inferior parietal lobule (*r* = .52, *p* = .002), right insula (*r* = .51, *p* = .003), median cingulate (*r* = .50, *p* = .003), fusiform gyrus (*r* = .51, *p* = .003), angular gyrus (*r* = .51, *p* = .003, calcarine fissure (*r* = .49, *p* = .004), and superior parietal gyrus (*r* = .50, *p* = .004). In addition, close association merely shows a significant negative correlation to the global efficiency of the left middle temporal gyrus (*r* = -.55, *p* = .001). The study also used the Brain Net Viewer [48] to make a 3D visualization of the results, shown in Fig 1.

**Table 3:** Brain Regions with Significant Correlations between Node Global Efficiency and Scores from Remote and Close Associative Tests.

Region	Category	X	Y	Z	*R*	*P*
Remote Association						
TPOmid.L	Temporal	-36.32	14.59	-34.08	0.66	0.001
IPL.R	Parietal	46.46	-46.29	49.54	0.52	0.002
INS.R	Subcortical	39.02	6.25	2.08	0.51	0.003
DCG.R	Frontal	8.02	-8.83	39.79	0.50	0.003
FFG.L	Temporal	-31.16	-40.30	-20.23	0.51	0.003
ANG.R	Parietal	45.51	-59.98	38.63	0.51	0.003
CAL.R	Occipital	15.99	-73.15	9.40	0.49	0.004
SPG.R	Parietal	26.11	-59.18	62.06	0.50	0.004
Close Association						
MTG.L	Temporal	-39.88	15.14	-20.18	-0.55	0.001

Note: R: right hemisphere; L: left hemisphere; TPOmid: Temporal pole: middle temporal gyrus; IPL: Inferior parietal, but supramarginal and angular gyri; INS: Insula; DCG: Median cingulate and paracingulate gyri; FFG: Fusiform gyrus; ANG: Angular gyrus; CAL: Calcarine fissure and surrounding cortex; SPG: Superior parietal gyrus; MTG: middle temporal gyrus.

## Discussion

This study first discriminated the differences in the connectivity structures of the white-matter with regard to remote and close associations at the level of the brain network and then discussed the correlations between the production of creativity and the operation of different regions of the brain. The results conform to the assumption that remote association has a significantly positive correlation to the connectivity efficiency of brain network structures. The global efficiency of the brain network shows positive connectivity to remote association. As such, the global efficiency represents the overall transmission ability of the brain network. When the network global efficiency is higher, the connecting paths between the nodes are shorter, and the signal transductions between all of the nodes in the network is faster. A positive correlation between the global efficiency and remote association means that when the transmission efficiency between organizations of the brain network is better, it is more likely that the individual will think of remote concepts. In addition, normalized local efficiency shows a significant negative correlation to remote association. The local efficiency represents the signal transmission abilities of different regions of the network, i.e., the clustering level of the neighboring nodes. When the local efficiency is higher, the nodes within a region of the network are more closely connected. The negative association between the normalized local efficiency and remote association means that when there are obvious connections in certain regional organizations of the brain network, it is difficult to produce remote concepts. The above results also support the idea that creativity is a complicated process that requires the integration of multiple perception functions [49].

On the other hand, the small-world properties of the network have a negative correlation to remote association. Different from a regular network with a higher clustering coefficient and a longer connecting path or a random network with a lower clustering coefficient and a shorter connecting path, a small-world network has both a better clustering coefficient and a shorter transmission path, which results in the effective mutual transmission of signals across the entire network and local parts of the network [32]. Because the index for small-world is the ratio between the normalized local efficiency [30, 32, 50] and the global efficiency and remote association is a positive correlation to the global efficiency and a negative correlation to local efficiency, the correlation between remote association and the small-world attribute is negative. In other words, when the network of the white-matter of the brain is a small-world network that combines the advantages of both the global and local connectivity efficiencies, it hinders an individual’s efficient production of remote concepts. Conversely, a random network with a comparatively low clustering coefficient and a shorter connecting path is more likely to produce remote concepts. This finding shows that remote association was not due to good global efficiency and local efficiency of brain networking, but was due to the long distance of the delivery across the regions. This finding supports the previous finding that divergent thinking positively correlated to global efficiencies of the relevant brain regions [27], but it does not support the finding by graph theory that divergent thinking negatively correlated with global efficiency in the female group [33]. The reason for the difference is the methods of structuring the network; the present study used the AAL-90 nodes network, which is different from the previous studies, which used a divergent thinking relevant nodes network [27] or an 83-node network [33], which led to different results.

This study further divided the brain into 90 regions of interest and considered each region of interest to be a node of the brain network. The study results show that the global efficiencies of the MTG and FG on the center left, the IPL on the right, insula, median cingulate, the AG, calcarine fissure, and the SPG have significantly positive correlations with remote association. This finding means that the above-mentioned regions are important hubs for signal transmission in the brain network, during which each individual region connects the remote concepts. In particular, this result conforms to the idea that was proposed in an existing study, that the temporal lobes play an important role in the production progress of remote concepts [17]. A study on fMRI noted that the fluent and unique performance of the divergent thinking of an individual is related to the activation of the MTG [18, 51, 52, 53, 54, 55], which indicates that the operation of the MTG has a significant correlation to the production of large numbers of novel ideas. In addition, Ellamil recorded the activities of brain nerves during the production of ideas by the participants and also discovered that the MTG and IPL are obviously activated during the production of creative ideas by the participants [18]. Furthermore, existing studies also determined that the functioning of the IPL, AG, and FG significantly influence the individual’s creativity [17, 56, 57]. The AG and FG are regarded as having the cognitive functions of responsible extraction of semantics [58] and word recognition [59], respectively. Moreover, the operation of the insula also influences the semantic processing level, mainly in the fluency of the production of words [60, 61, 62]. Additionally, the aforementioned regions distribute over both cerebral hemispheres, which means that creativity requires the cooperation of the right and left brains [23]. To summarize, most of the nodes of the brain network that are positively correlated to remote association are regions that are also related to the production of semantic or innovative ideas. This result also supports the connectivity theory, which proposes that creativity can be explained by of the production of remote concepts or semantics [1, 63].

On the other hand, close association also has a significant correlation to the connectivity efficiency of the white-matter network of the brain and is only negatively correlated with the global efficiency of the brain network. This finding means that the higher the overall transmission efficiency between the nodes of the brain network is, the more unlikely association it is that the individual will produce close concepts. Close association is not related to the local efficiency, and in small worldness, the development of close associations for the topics had no relationship with the region delivery capacity and attributes of the networking structure. Close association is not related to the standardized local efficiency and the attributes of small worldness; however, we found a tendency of positive correlation. The correlation shall be significant if the sample becomes larger in future studies. With respect to the global efficiencies of the nodes, only the MTG on the left shows a significantly negative correlation to the scoring for close association. This finding means that apart from the connections to remote concepts, the temporal lobes also influence the production of close concepts, and when the signal transmission of the MTG is better, it is more unlikely for the individual to produce creative close concepts. This result agrees with the above-mentioned discovery that remote association is a positive correlation to the MTG.

Interestingly, this study discovered that remote and close associations show completely opposite correlation conditions to the global efficiency of the brain network. Specifically, the global efficiency has a positive correlation to remote association but a negative correlation to close association, which shows that these two types of association have completely different connection methods in the brain network. Remote association is the ability to connect independent elements into new relationships [1], which refers to a process in which individuals generate all of the concepts and combine them into novel outputs. Therefore, the key point for the production of creative ideas is whether the semantic information can be extracted successfully. The spreading activation theory uses the interconnectivity methods of the network nodes to explain how an individual extracts semantic knowledge and discriminates whether it is a close concept or remote concept based on its relevance to the theme and the length of the connecting path. When the connecting path between the semantic network and the theme is a connecting path that is relatively far and the relevance is low, the knowledge node is considered to be a remote concept. In contrast, close concepts are characterized by a short path to the theme and a high level of relevance. Furthermore, compared to close concepts, remote concepts are not easy for individuals to connect [64]. Analysis of the graph theory of this study indicates that an individual’s association to remote concepts is the result of the effective transmission between the different nodes of the brain network. Specifically, remote association requires the simultaneous operation of multiple brain regions. In contrast, when the overall transmission efficiency of the network is better, it is more unlikely to produce close concepts, which means that close association requires the activation of only a few brain regions.

Notably, unlike previous studies that used fMRI to explore the activation situations of the brain under certain operations, this study used diffusion tensor imaging during the quiescent state and graph theory to analyze the correlations between the connectivity efficiencies of the network structure of the white-matter of the brain and the ability to perform remote association. The analysis results show that the intrinsic connectivity of the brain structure in a quiescent state is a significant predictor of creativity.

The present study has limits and suggests continued research. First, existing studies believe that the accuracy of the analysis index of the brain graph theory is closely related to the resolution of the network (ex: the number of nodes) [29, 65]. Future studies can increase the regions of interest to 1024 [46, 66] to enhance the validity of the analysis results of the network. Second, the local efficiency and clustering coefficient had no correlation to remote association or close association; after standardization, however, the correlations became significant. These findings show that there were still some confounding variables that were uncontrolled (e.g., the speed of processing, perceptual tissues); except for the network nodes, some attributes, such as the node degree, node betweenness and so on, are worthwhile to discuss further. Third, remote association is also an ability of insightful problem solving, because the experience of “aha” occurs for individuals during both tasks [67], and both tasks have a medium correlation [68]. Remote association can occur through an insight process or non-insight process [69], but only the former involves the “aha” experience. Past research has indicated that the strength of the “aha” was related to the time (length) of incubation [70], and the time for remote association and the time for close association were different [14], which might be the reason for the difference in the “aha” experience. This finding suggests that future research could compare the “aha” experience between the two association tasks.

## Conclusions

The present study used a topological perspective to build a brain network with 90 nodes for regions of interest and investigated the connection efficiency of the brain white matter with remote association and with close association. We found that the integrated efficiency of delivery for brain networking was different between remote association and close association, and better across-region delivery helps remote association, indicating that global efficiency is critical to individuals for remote association and close association. Additionally, low clustering coefficients and a random network of short connection paths were helpful to the development of remote associations, which indicates the influence of connectomics of brain networking on remote association and close association. This finding supports the statement that creativity requires cooperation among several brain regions [26, 27, 28, 71, 72, 73].

## Supporting Information

S1 File This file include participants’ number (i.e. ID), age, gender, VIQ, remote and close association scores (i.e. Remote, Close), brain network properties (i.e. Cp, Lp, gE, locE, Sigma, Gamma, Lambda, and n01 –n90). (XLSX)
