# Correction: Discriminating the Difference between Remote and Close Association with Relation to White-Matter Structural Connectivity

**DOI:** 10.1371/journal.pone.0170864

**Published:** 2017-01-20

**Authors:** Ching-Lin Wu, Suyu Zhong, Hsueh-Chih Chen

The first author’s name is spelled incorrectly. The correct name is: Ching-Lin Wu. The third author’s name is also spelled incorrectly. The correct name is: Hsueh-Chih Chen. The correct citation is: Wu C-L, Zhong S, Chen H-C (2016) Discriminating the Difference between Remote and Close Association with Relation to White-Matter Structural Connectivity. PLoS ONE 11(10): e0165053. doi:10.1371/journal.pone.0165053.

There is an error in affiliation 1 for authors Ching-Lin Wu and Hsueh-Chih Chen. Affiliation 1 should be: Department of Educational Psychology and Counseling, National Taiwan Normal University, Taipei, 10610, Taiwan.
